# Effect of Process Parameters on Welding Residual Stress of 316L Stainless Steel Pipe

**DOI:** 10.3390/ma17102201

**Published:** 2024-05-08

**Authors:** Xiaowei Jiang, Wenhui Wang, Chunguang Xu, Jingdong Li, Jiangquan Lu

**Affiliations:** 1 School of Mechanical Engineering, Jiangsu University of Science and Technology, Zhenjiang 212003, China; 2 School of Foreign Languages, Jiangsu University of Science and Technology, Zhenjiang 212003, China; snowshine561@163.com; 3 Key Laboratory of Fundamental Science for Advanced Machining, Beijing Institute of Technology, Beijing 100081, China; xucg@bit.edu.cn; 4 Jiangsu Institute of Automation, Lianyungang 222061, China; mengdongzhu@163.com (J.L.); work716@163.com (J.L.)

**Keywords:** 3D finite element, welding energy, welding speed, welding start position

## Abstract

316L stainless steel pipes are widely used in the storage and transportation of low-temperature media due to their excellent low-temperature mechanical properties and corrosion resistance. However, due to their low thermal conductivity and large coefficient of linear expansion, they often lead to significant welding residual tensile stress and thermal cracks in the weld seam. This also poses many challenges for their secure and reliable applications. In order to effectively control the crack defects caused by stress concentration near the heat-affected zone of the weld, this paper establishes a thermal elastoplastic three-dimensional finite element (FE) model, constructs a welding heat source, and simulates and studies the influence of process parameters on the residual stress around the pipeline circumference and axial direction in the heat-affected zone. Comparison and verification were conducted using simulation and experimental methods, respectively, proving the rationality of the finite element model establishment. The axial and circumferential residual stress distribution obtained by the simulation method did not have an average deviation of more than 30 MPa from the numerical values obtained by the experimental method. This study also considers the effects of welding energy, welding speed, and welding start position on the pipe’s circumferential and axial residual stress laws. The results indicate that changes in welding energy and welding speed have almost no effect on the longitudinal residual stress but have a more significant effect on the transverse residual stress. The maximum transverse residual stress is reached at a welding energy of 1007.4~859.3 J/mm and a welding speed of 6.6 mm/s. Various interlayer arc-striking deflection angles can impact the cyclic phase angle of the transverse residual stress distribution in the seam center, but they do not alter its cyclic pattern. They do influence the amplitude and distribution of the longitudinal residual stress along the circumference. The residual stress distribution on the surface of the pipe fitting is homogenized and improved at 120°.

## 1. Introduction

316L is a kind of austenitic stainless steel containing a small amount of ferrite. Because of its good weldability and corrosion resistance, it is often used for chemical storage, low-temperature medium transportation, and other occasions [1,2,3,4,5]. In the welding of 316L thin-walled pipes, hot cracks can be easily produced, so filling welding becomes a necessity. Filler welding is usually composed of multiple bead layers. The large amount of heat input brings high residual stresses to the heat-affected zone. Once these residual stresses exceed the yield strength of the material, they will cause stress release and deformation, which further lead to stress corrosion, fatigue cracking, and other leakage accidents.

Pipe welding stress control has become a popular research topic in recent years. In the past, scholars have studied welding stress and strain using experimental methods or simplified numerical calculations. However, with the emergence of thermoelastic–plastic finite element analysis and continuous improvements in computer performance, numerical simulation using finite element methods has become a widely respected approach among researchers. To ensure the reliability of 316L stainless steel pipes in resisting low-temperature impact during use and to obtain good weld tissue performance, scholars worldwide have studied the influence of process parameters such as welding speed, welding power, welding sequence, interlayer temperature, and preheating temperature on residual stresses and the degree of deformation of the pipe fitting’s structure. They have proposed reducing post-weld stress concentration through the optimal selection of process parameters [6,7,8,9,10].

To improve simulation accuracy and computational efficiency, some scholars have applied neural network training for heat sources, simplified models with rotational or axial symmetry, and developed thermal–mechanical simulation methods that consider solid phase transitions and softening effects. They have also developed stress simulation processing methods. I. Sattari-Far [11] proposed a three-dimensionalentralomechanical analysis method to study single-pass pipe welding in nine different welding sequence conditions. The appropriate welding sequence can effectively reduce welding deformation. Navid [7] utilized the heat source model parameters acquired through neural network training on the 316L pipe, based on the one-quarter circumferential circle and the direction of the welding simulation and experimental validation. The results showed that when using full circumferential welding, residual stresses and deformations are distributed more uniformly along the circumferential direction. Zhao et al. [12] also investigated this phenomenon. Their study analyzed the impact of heat input, bevel form, and the number of weld beads on residual stresses during the welding of T92 and S30432 dissimilar steels. The results showed that reducing the slope angle reduced the axial and circumferential residual stresses. Additionally, the peak of the residual stresses on the tensile side of S30432 decreased with a smaller heat input and an increased number of weld beads. Kohandehghan et al. [13] investigated the distribution of residual stresses in welded joints under the coupled state of welding sequence and welding fixture constraints. Abid et al. [14] conducted a three-dimensional thermal–mechanical analysis to investigate the effect of the mechanical–thermal coupling state of pipeline flange joints on weld deformation and the residual stress state. The results were found to be closer to the actual state of stresses. In addition, Gharib et al. [15] studied the effects of the clamping constraint position and the clamping holding time on weld deformation and the residual stress distribution of 304L austenitic stainless steel butt-welded joints throughentralomechanical analyses, revealing that the state of the external constraint position and timing are also key factors influencing weld residual stresses.

In recent years, methods such as optimizing heat input, improving the accuracy of heat source parameters, and simplifying simulation models have been widely used in finite element simulation analysis of the effects of welding sequence, heat input, groove form, and the number of weld bead layers on the residual stress and deformation in pipeline welding. However, due to the irrationality of the symmetry assumption and the over-simplification, the research and analysis scope is limited, and only the influence of process changes on the pipeline stress in a fixed cross-section can be obtained. This limitation makes it difficult to effectively guide the process optimization and stress and deformation control of conventional pipe fitting welding production [16,17,18,19,20,21,22,23,24,25,26,27].

In order to deeply study and master the overall distribution law of residual stress after multilayer welding of 316L pipes under process parameters, this paper first establishes a finite element model based on the morphology characteristics of the butt weld and verifies the rationality of the three-dimensional finite element model through experimental methods combined with process parameters. In addition, controllable welding energy, welding speed, and welding start position of the three process parameters are also selected as the object, respectively, to study the rule of change in pipeline circumferential and axial welding residual stresses under the different factors mentioned above. This study analyzes the correlation and influencing characteristics of various factors on the overall stress distribution of multilayer welded pipe fittings, which are of great significance for the coordinated control of welding residual stresses [28,29,30,31,32].

## 2. Experimental Analysis

### 2.1. Experiment Material Selection

The weld base metal selected for this study is SUS316L. GMS-316L solid core stainless steel wires of size 1.2 mm are used, with the base metal and wire chemical composition shown in Table 1. Test samples with a diameter of 168 mm, length of 600 mm, and wall thickness of 8 mm and 5 mm on both sides of a 35° V-butt-welded stainless steel pipe are selected, with a 1 mm weld root assembly gap. Argon, with a purity of 99.99%, is used as a shielding gas. The welding process is carried out with argon inside and outside the weld.

### 2.2. Experimental System

**Figure 1:**
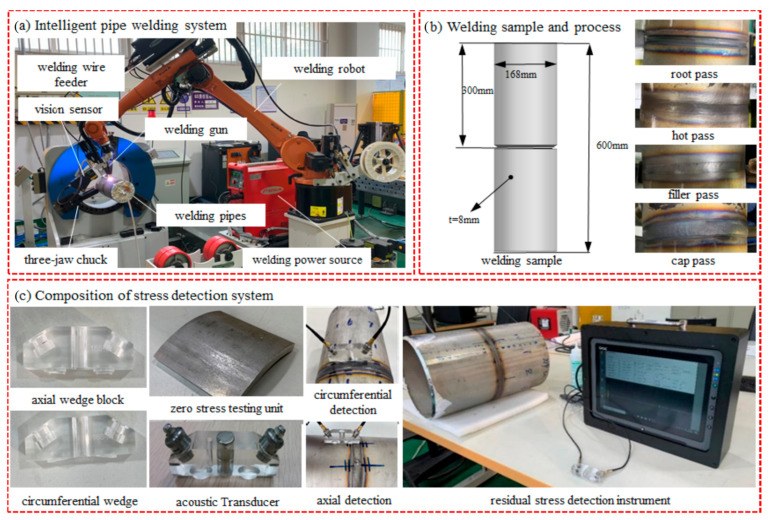
Welding systems, specimen preparation, and testing methods.

The experimental system in this study comprises three components: a smart pipe welding robot system, a welded sample pipe, and a residual stress detection system. The intelligent pipe welding robot system employs a KUKA KR16 six-axis industrial robot (KUKA, Augsburg, Germany), a Fronius Trans Tig 5000 Job G/F TIG power supply (Fronius, Welles, Austria), a KB370 fully automatic rotary three-jaw chuck (Huaheng, Xuzhou, China), and a BU-01 residual stress ultrasonic detector (BIT, Beijing, China). The system comprises an ultrasonic transducer, an ultrasonic signal excitation card, an ambient temperature sensor, a signal collector, a calibration specimen, etc. Its detection range is from −1000 mPa to +1000 mPa, with an error margin of ±30 mPa. The system uses a one-receiving-one-transmitting mode for single-point online acquisition. The system conforms to the national standard GB/T 32073-2015 [33] and can detect residual stress below the surface of the component at a certain depth. It can also detect residual stress distributed along the thickness or depth direction of the component. To enable the ultrasonic sensor to be coupled with the structural surface, the axial and circumferential wedge can be designed and manufactured to fit a pipe diameter of 168 mm. Additionally, 100 × 100 zero-stress specimens can be produced using the GB/T25712 [34] vibration time-distortion method for raw material specimens after treatment. Figure 1 shows a schematic diagram of the pipe fitting for axial and circumferential detection.

### 2.3. Experimental Method

High-precision rotary cut pipe butt welding is used as the test object. The butt gap is 1 mm, and the alignment assembly is completed before welding to ensure that the amount of misalignment is less than 0.5 mm. The assembly weld area and weld circumference are wiped down using mechanical grinding and acetone to remove surface oils and oxidized layers. The process involves applying root pass, two layers of filling passes, and one layer of cover pass using Gas Tungsten Arc Welding (GTAW). The process is detailed in Table 2.

The residual stress ultrasonic detector is used to detect the pipe stress after welding, and the axial and circumferential signal collectors are selected to obtain the axial and circumferential residual stresses through wedge coupling.

## 3. FE Simulation

A three-dimensionalentralomechanical coupling finite element model for butt-joint argon arc welding of pipes is established based on SYSWELD. Using the nonlinear heat conduction model, we first solve for the temperature field distribution and its time-domain variation data in the pipe fitting space. Then, we obtain the residual stresses based on the nodal temperatures as the boundary condition inputs. These residual stresses are used as input conditions for mechanical elasticity and plasticity calculations.

### 3.1. Finite Element Model

**Figure 2:**
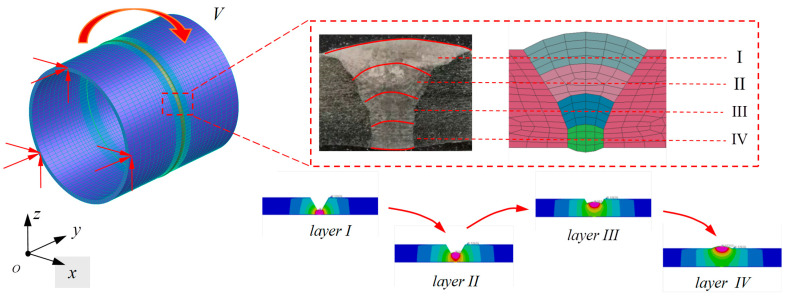
Finite element modeling of pipe multilayer welding.

The finite element model of single-layer multi-pass butt welding with a V-bevel pipe is constructed, and four welds are set with reference to the macroscopic morphology of actual welds, as shown in Figure 2. The total length of the pipe is 600 mm, the outer diameter is 168 mm, and the wall thicknesses are 8 mm and 5 mm. Considering that the temperature gradient near the welding heat source varies a lot, in order to improve the computational efficiency and obtain better computational accuracy, the principle of gradually decreasing the mesh density of the seam center, heat-affected zone, and peripheral area is adopted, but the element sizes along the direction of the weld beads are kept the same, close to 4 mm, and the minimum element size is 1.267 mm in the direction of the pipe axis.

### 3.2. Heat Source Modeling and Input Parameters

**Figure 3:**
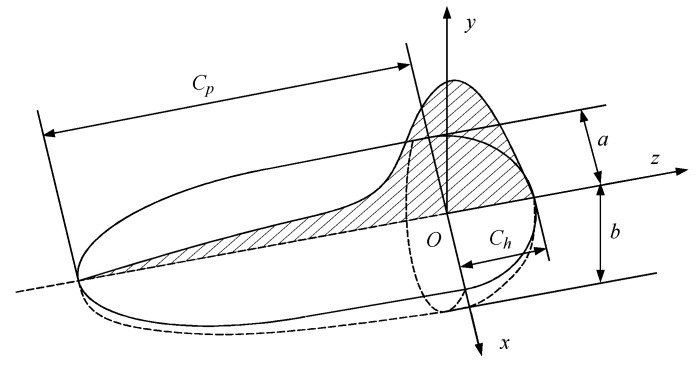
Double ellipsoidal heat source model.

In combination with the characteristics of the argon arc welding process, the heat source takes the form of a double semi-ellipsoid with front and back asymmetry, as shown in Figure 3. The parameters of the long and short axes of the two semi-ellipsoids are defined as ($c_{h}$, $c_{p}$, a, and b), and the proportion of heat in the front and rear parts is represented by $f_{h}$ and $f_{p}$, respectively. The settings for the shape parameter are presented in Table 3. The heat flow distribution of the heat source can be expressed by the following equation:

$$q_{h}(x,y,z)=\frac{6\sqrt{3}f_{h}Q}{abc_{p}\pi \sqrt{\pi }}\exp(-\frac{3x^{2}}{a^{2}}-\frac{3y^{2}}{b^{2}}-\frac{3z^{2}}{c_{h}^{2}}),x\geq 0$$

$$q_{p}(x,y,z)=\frac{6\sqrt{3}f_{p}Q}{abc_{p}\pi \sqrt{\pi }}\exp(-\frac{3x^{2}}{a^{2}}-\frac{3y^{2}}{b^{2}}-\frac{3z^{2}}{c_{p}^{2}}),x<0$$

where $f_{h}+f_{p}=2$, $f_{h}=1.2$, and $f_{p}=0.8$.

Referring to the welding energy formula, the expression for the energy obtained from the heat source per unit length of weld during fusion welding can be derived as follows:

$$P=\eta P_{0}=\eta UI$$

where U is the voltage (V), I is the welding current (A), $P_{0}$ is the total welding arc power (J/s), and η is the electricity heat conversion efficiency (AC tungsten arc welding thermal efficiency is generally 0.68–0.85; this paper takes 0.8).

### 3.3. Thermal Elastic–Plastic Simulation

The strain in the thermoplastic process comprises elastic strain $d\varepsilon_{e}$, plastic strain $d\varepsilon_{P}$, and thermal strain $d\varepsilon_{T}$. The complete expression for the strain increment under thermoplastic deformation can be obtained as follows:

$$d\varepsilon =d\varepsilon_{e}+d\varepsilon_{P}+d\varepsilon_{T}$$

**Figure 4:**
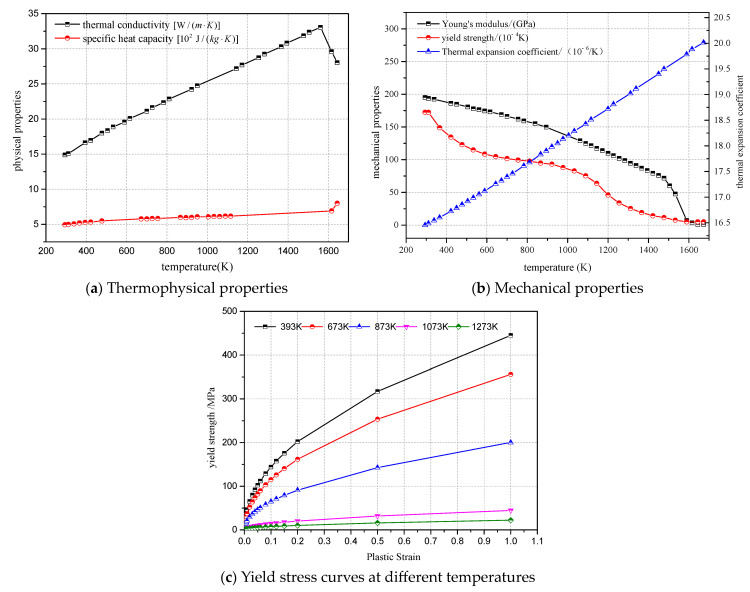
Thermal, physical, and mechanical properties of 316L stainless steel.

The thermal process of welding is characterized by a sharp increase in temperature and a complex process. To accurately simulate the dynamics of the temperature field process, the material selected here is 316L stainless steel for circumferential butt-welded joints. The relevant thermophysical parameters of the 316L material are obtained, among which are the following: density of 7720 (kg/m^3^), thermal conductivity [W/(m·K)], specific heat capacity [10^2^ J/(kg·K)], coefficient of thermal expansion (10^−6^/K), Young’s modulus (Gpa), Poisson’s ratio, and yield strength and strain as a function of temperature. The relationship is shown in Figure 4.

After dividing the established three-dimensional model of the pipe butt weld bead into finite elements, external constraints and temperature fields are applied. The welding energy and welding speed for each bead are determined based on the welding multilayer multi-bead welding information. Material properties are considered along with changes in temperature and other disturbing factors. The temperature increment is then calculated after iteration. The node temperatures from the thermal analysis are imported and used in the stress analysis to obtain the stress distribution results during and after welding, while maintaining the same units and nodes as the thermal analysis.

### 3.4. Simulation Scheme

This study focuses on three parameters that have a significant impact on residual stresses: welding energy, welding line speed, and welding start position. To ensure reliable welding results, consistent bottoming welding process parameters are used. The process parameters are varied during filling and cosmetic welding to compare their post-weld residual stresses. Two types of pipe fittings are separately selected based on their diameter and wall thickness (168 mm and 8 mm; 5 mm). A single-pass multilayer welding process is used to create 11 simulation models, divided into three independent comparison groups (*a*, *b*, and *c*), with the relevant parameters detailed in Table 4.

Group *a* analyzes the effect of different welding energy inputs on the temperature field and stress distribution of pipe fittings using the same mesh model and process welding speed. The distribution characteristics of residual stresses in the weld are analyzed by 4 groups with different energy inputs.

Group *b* analyzes the effects of different welding speeds on the temperature and stress fields of pipe fittings while maintaining the same structural characteristics, external constraints, and process welding energy input. This study analyzes the effect of energy input on the distribution of welding residual stresses using four different welding speed inputs.

**Figure 5:**
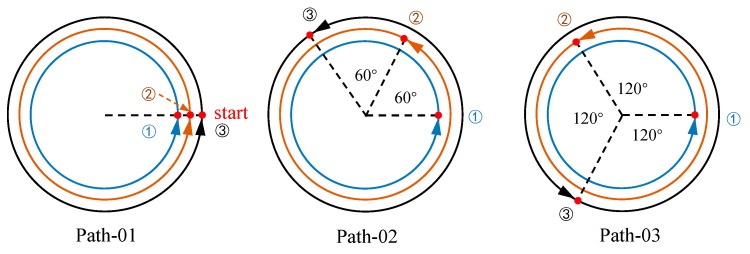
Setting of interlayer arc-striking deflection angles.

Group *c* studies the effects of temperature field and stress field distribution with different welding start positions. Under the condition of the same welding energy input and welding speed, three groups of different interlayer deflection angles are established to study its effect on welding residual stress, as shown in Figure 5.

## 4. Results and Analyses

### 4.1. Comparative Validation of Simulation and Test

**Figure 6:**
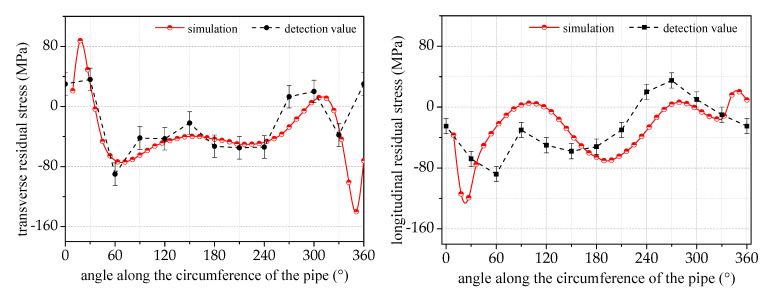
A comparison of the simulated and measured residual stresses at 20 mm from the center of the circumferential joint.

We select group *a*_2_ of the welding process parameters as the experimental base condition for the simulation and welding process testing; after the completion of welding to meet the national standard GB/T 32073-2015, a residual stress ultrasonic detector at a distance from the center of the circumferential joint of 20 mm, respectively, is used to detect the transverse and longitudinal residual stress. The results of the finite element simulation compared with the experimental results are shown in Figure 6.

It is evident from the comparison chart between the detection and simulation results that the distribution of residual stress along the circumference of the pipe follows a converging pattern. Although there is some numerical deviation, it is not significant, with an average deviation of no more than 30 MPa. A significant deviation is observed at the beginning of the weld, where the value is close to 100 MPa. This is mainly due to the striking and ending arc points of the weld; the actual welding current at this position is in an unstable state and the actual energy value will be smaller than the simulation value.

**Figure 7:**
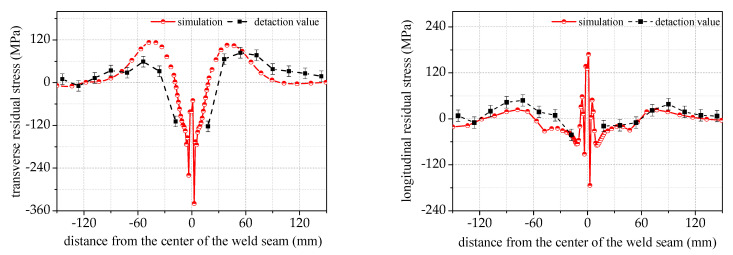
A comparison of the simulated and measured residual stresses on the surface of the pipelines in the axial direction.

Figure 7 shows a comparison between the experimental and simulation results of stress in the axial direction. The trend of the measured and simulated stress values is similar, with an average deviation in the transverse and longitudinal residual stresses of no more than 30 MPa. The stress on the side far away from the weld has a smaller deviation than the stress in the near-weld region. This stress difference is due to the large gradient near the weld region. It is important to note that even a slight change in the measurement position can result in a significant difference in stress.

Based on the comparison between the actual measurements and simulations, deviations in measurements may occur in the weld start and finish points and near the weld region due to the large stress change gradient. Despite this, the overall trend of the measurement results remains consistent.

### 4.2. Effect of Welding Energy on Residual Stress Distribution

**Figure 8:**
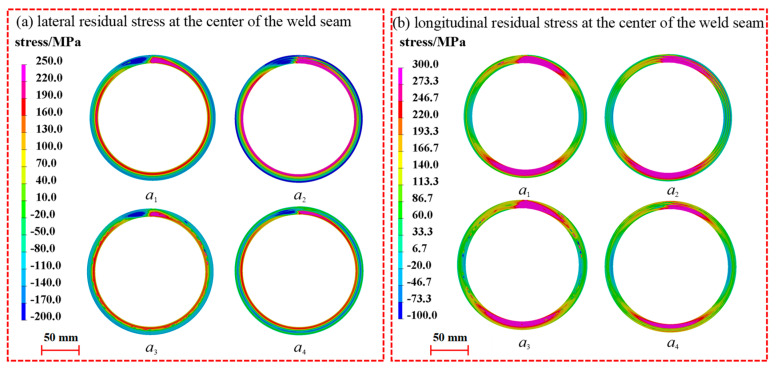
A comparison of residual stresses in the circumferential circle of the seam center.

Figure 8 shows that the residual stresses in the seam center profile have similar distribution trends for the four welding energy inputs, but the effects of different welding energy inputs vary considerably in the heat-affected zone. The transverse residual stresses indicate that the inner ring is generally under tension, while the outer ring is under compression. The inner ring exhibits significant stress concentrations and gradients in the transverse residual stresses near the arc-striking and ending points. Tensile stresses are predominantly near the arc-striking point, while compressive stresses are predominantly at the arc-ending point. From the four groups of comparisons, it can be observed that as the welding energy input increases, so does the stress gradient. However, there is a certain point at which further increases in the welding energy input will not have any significant effect on the circumferential stress distribution trend. The longitudinal residual stress is concentrated on the welding arc-striking side of the local area and entralral symmetrical region, resulting in a large residual tensile stress. The higher the welding energy, the more concentrated the longitudinal residual stresses, and the larger the area or range they affect.

The transverse residual stress trend on the pipe surface along the axial direction reflects the overall stress distribution on the surface of the pipe fittings after welding. The center area of the weld is the stress concentration area, mainly exhibiting the largest transverse compressive stress and the largest longitudinal tensile stress. In the heat-affected zone of the weld, the transverse residual stress transitions gradually from the center of compressive stress to transverse tensile stress, while the longitudinal residual stress transitions from tensile stress to compressive stress.

**Figure 9:**
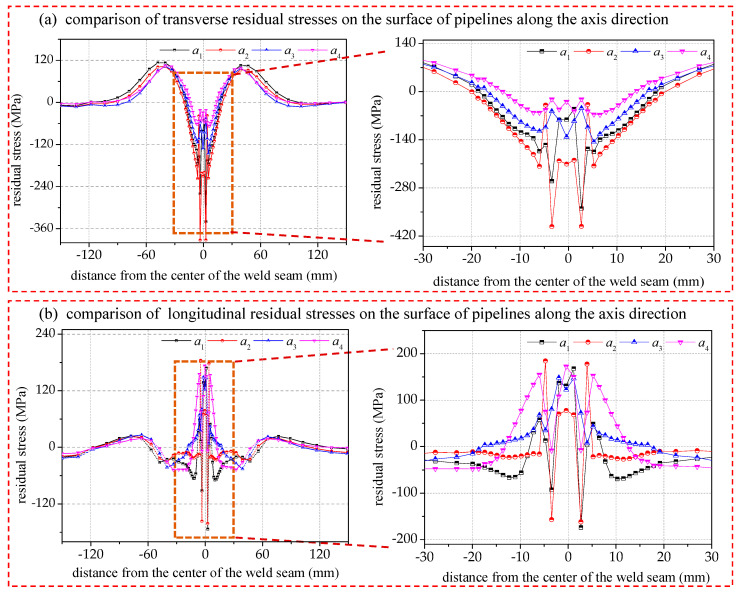
Effect of welding energy input on distribution of residual stresses on pipe surfaces in axial direction.

Figure 9 shows that a larger welding energy input increases the area of influence of the transverse residual stresses. However, the stress amplitude does not significantly increase due to the constraints of the fitting structure. Instead, the concentration of residual stresses is homogenized due to the distant constraints, resulting in a slightly smaller stress amplitude of *a*_1_ compared to *a*_2_. The longitudinal residual stresses are minimally affected by the welding energy outside of the heat-affected zone and only slightly affected within the adjacent area. The comparison graph shows that the groups *a*_1_ and *a*_2_ have the highest residual tensile and compressive stresses in the transverse and longitudinal directions, respectively. As the heat-affected zone expands and the welding energy output reaches a certain value, the surrounding structures become involved in stress homogenization, resulting in a decrease in the local stress amplitude. The critical value of the welding energy is between 1007.4 and 859.3 J/mm.

**Figure 10:**
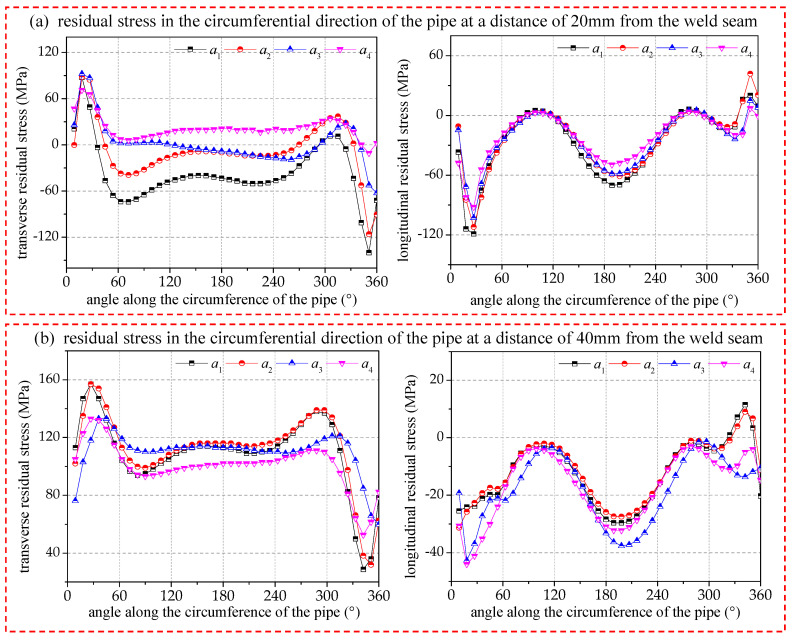
Comparison of residual stresses of pipe circumference at 20/40 mm from seam center.

Figure 10 gives a comparison of the transverse and longitudinal residual stresses on the surface of the pipe circumference at distances of 20 mm and 40 mm from the center of the circumferential joint, respectively. The overall magnitude and nature of the transverse residual stresses will be different for pipe circumferences with different distances from the center of the circumferential joint. In the region near the heat-affected zone (20 mm from the center of the circumferential joint), after the arc-striking point, the local area is dominated by tensile stress, before the ending point, the local area is dominated by compressive stress, and the vast majority of the intermediate area exists in a lower tensile or compressive stress state. The specific tensile or compressive stress mainly depends on the magnitude of the welding energy. In the heat-affected transition region (40 mm from the center of the circumferential joint), the majority of tensile stresses are manifested, and only in the vicinity of the arc-closing point are compressive stresses manifested. In addition, due to the influence of structural constraints, the transverse residual stresses will become larger with the welding energy increase, but there is a critical value. When the welding energy reaches 693.4 J/mm, the energy input increases again, and the transverse residual stresses no longer increase.

The longitudinal residual stresses are basically the same in the overall distribution trend, with the seam center mainly showing longitudinal tensile stresses and the heat-affected zone mainly showing longitudinal compressive stresses. Moreover, the longitudinal residual stress has a periodic fluctuation, with large values obtained at 0–30° and 180–210°, and minimum values near 90–120° and 270–300°, respectively. The distribution of the longitudinal residual stresses in the circumference at different distances from the ring seam also has similar periodic fluctuations as those at the center of the circumferential joint, but its fluctuation pattern is little affected by the energy input.

### 4.3. Effect of Welding Speed on Residual Stress Distribution

**Figure 11:**
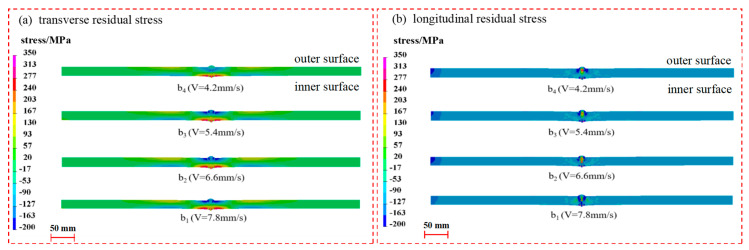
Effect of welding speed on transverse and longitudinal residual stresses in pipe sections.

Figure 11 shows that for transverse residual stresses, the distribution of the outer and inner surfaces near the seam center and the heat-affected zone is contrasting. The stress gradient of the cross-section increases as the welding speed increases. However, there is no significant change in the stress gradient when the welding speed reaches 6.6 mm/s. The distribution trend of the longitudinal residual stress is not directly related to welding speed. The longitudinal residual stress does not significantly change in the seam center and the heat-affected zone with changes in speed.

**Figure 12:**
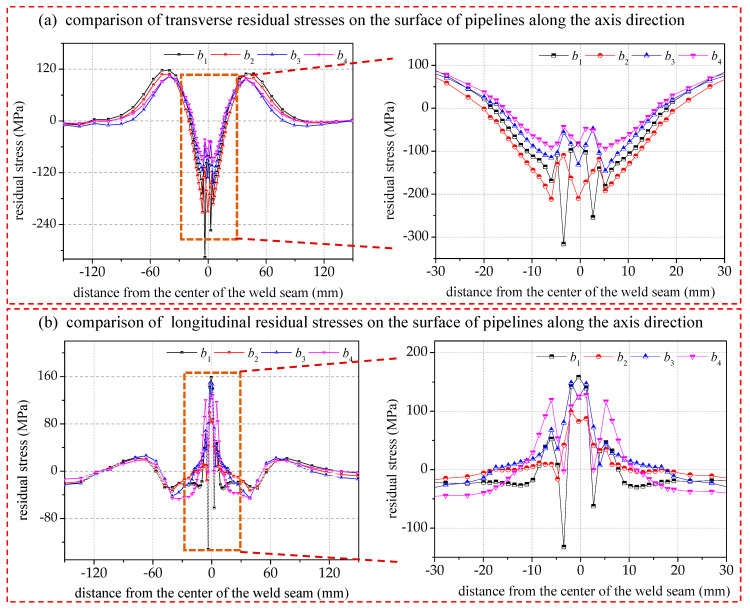
Effect of welding speed on residual stress distribution on pipe surface along axial direction.

The maximum transverse compressive stress and longitudinal tensile stress appear in the seam center, and as the distance from the weld center is increased, the transverse compressive stress and longitudinal tensile stress in the heat-affected zones on both sides of the weld gradually decrease, and the reversal occurs at about 20 mm, as shown in Figure 12. Subsequently, the stress becomes transverse tensile stress and longitudinal compressive stress. And then, the transverse tensile stress no longer reverses, and the longitudinal compressive stress reverses again to longitudinal tensile stress at about 60 mm.

In general, the change in welding speed by affecting the size of the heat-affected zone makes the range of stress concentration in the weld change somewhat but does not affect the overall distribution pattern. When the welding speed is faster, the fixed line energy is output in a shorter time, making the power per unit time larger. However, due to the limited heat exchange rate of the material, the heat-affected zone will become smaller, while the gradient of the transverse and longitudinal residual stresses will be larger in the weld center region. If it is farther away from this region, the effect will be smaller. When the welding speed decreases near the seam center, there should be an extreme value of the transverse residual compressive stresses, which is obtained when the welding speed is between 4.2 mm/s and 5.4 mm/s.

**Figure 13:**
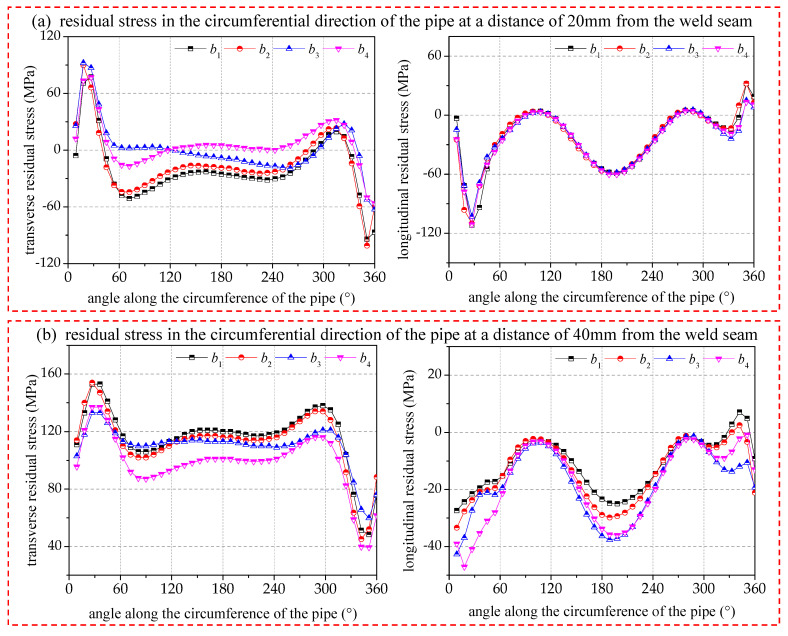
A comparison of the residual stresses of the pipe circumference at 20/40 mm from the seam center.

From the comparison of the transverse residual stresses at different distances from the center of the circumferential seam in Figure 13a (i.e., at 20 mm and 40 mm), it is observed that the higher the welding speed, the higher the transverse residual tensile or compressive stresses, and the deviation at the nearer distance is significantly larger than that at the farther distance. This indicates that the amplitude of the welded transverse residual stresses in the heat-affected region is positively correlated with the welding speed, and the influence coefficient is inversely correlated with the distance from the seam center.

A comparison of the longitudinal residual stresses at different distances from the center of the circumferential seam is given in Figure 13b. Except for the obvious difference in the longitudinal residual stress at the center of the circumferential seam, the longitudinal residual stresses at distances of 20 mm and 40 mm are less sensitive to the welding speed and have lower values. At the center of the circumferential seam, the longitudinal residual stresses are cyclically varying tensile stresses, and the positions of the 0° angle and the 180° angle are the stress concentration areas, where the higher the welding speed, the greater the longitudinal residual tensile stresses.

### 4.4. Effect of Interlayer Welding Start Position on Residual Stress Distribution

The interlayer welding start position differs from other process parameters in that it does not substantially change the amount of power or energy input to the welding process. It is only adjusted by changing the striking position of the weld bead. A pipe with a diameter of 168 mm and a wall thickness of 5 mm is used here to investigate the residual stress distribution characteristics for three different interlayer welding start positions. To ensure the consistency of the input parameters, the same weld bead, line energy input, and welding line speed are used.

**Figure 14:**
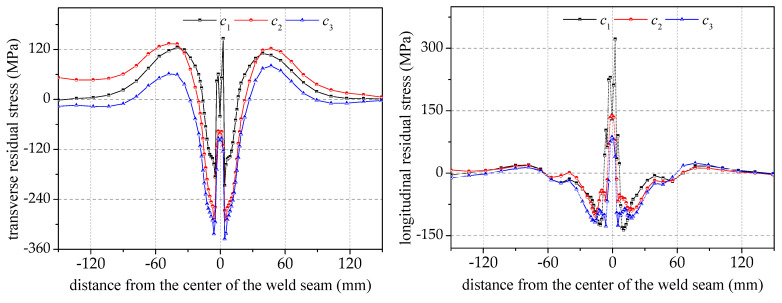
Effect of interlayer arc-striking deflection angle on axial residual stress distribution.

Figure 14 compares the distribution of weld residual stresses on the axial surface of the pipe fittings at deflection angles of 0°, 60°, and 120°. For transverse residual stresses, when the interlayer arc-striking deflection angle is 0°, the transverse residual stresses are maximal in the center region of the weld, while the compressive stresses in the heat-affected zone are minimal. The residual stress in the transverse direction is minimal at a 120° deflection angle, with the seam center experiencing tensile stress and the heat-affected zone experiencing compressive stress. In terms of the longitudinal residual stress, the seam center zone experiences the maximal tensile stress at a 0° interlayer welding start position, while the minimal tensile stress is observed at a 120° deflection angle. There are no significant differences in the longitudinal residual stresses between the heat-affected zone and its peripheral regions.

**Figure 15:**
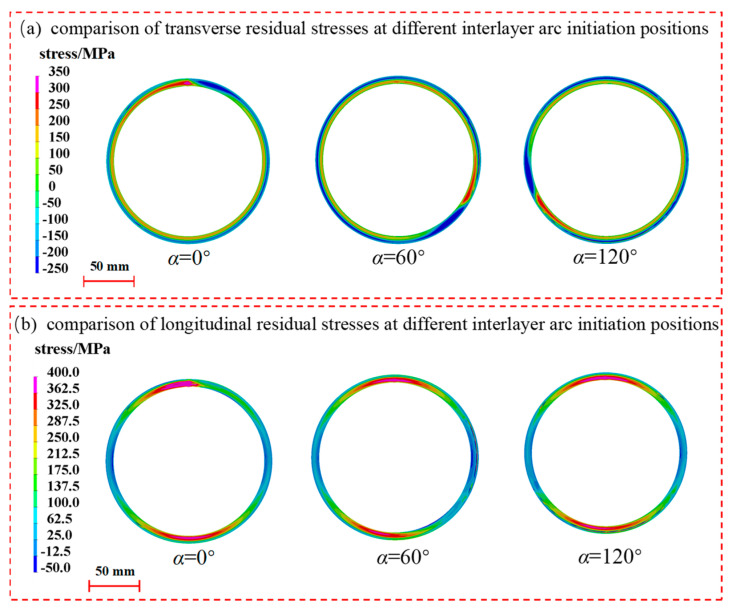
Effect of interlayer arc-striking deflection angle on transverse and longitudinal residual stresses in seam center.

**Figure 16:**
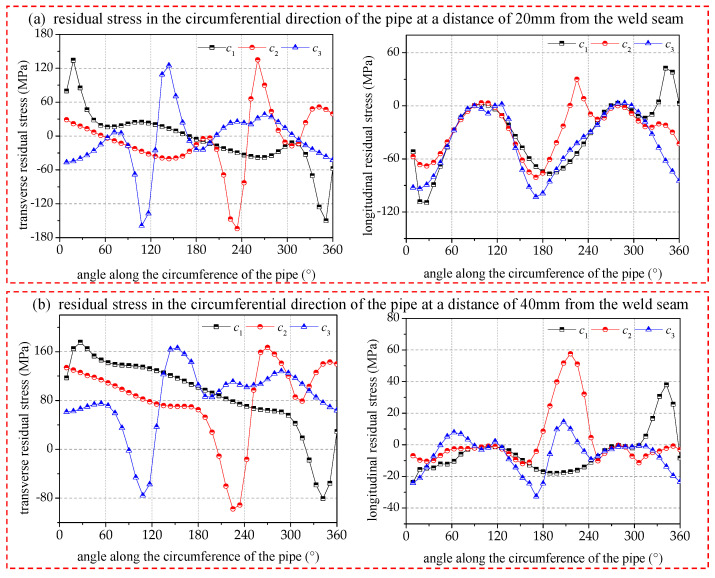
A comparison of the residual stresses of the pipe circumference at 20/40 mm from the seam center.

Different interlayer welding start positions can affect the deflection of the transverse residual stress in the circumference of the seam center, and it can be seen from Figure 15 that the transverse residual stress in the seam center rotates by an angle as large as the deflection angle of the interlayer welding starting position. The stress gradient and amplitude changes are not obvious. And from the transverse residual stress distribution at 20 mm and 40 mm in Figure 16, it can be clearly observed that the interlayer deflection angle will change the cyclic phase angle of the stress distribution but will not change its cyclic law.

The comparison graph shows the longitudinal residual stresses at different distances from the seam center. It is evident that the welding start position has a minimal impact on the amplitude and distribution of the longitudinal residual stresses along the circumference. However, it does affect the distribution of the longitudinal residual stresses at distances of 20 mm and 40 mm. The arc-striking deflection angle of 120° experiences minor fluctuations around the circumference, but the amplitude remains relatively stable. The reduction in heat input has homogenized and improved the distribution of surface residual stress away from the weld area.

## 5. Conclusions

This study establishes a finite element model of butt welding for 316L stainless steel pipes. A heat source model is constructed, and the simulation results are verified using a residual stress ultrasonic detector. The results confirm that the simulation method can replace the actual measurement method to a certain extent. The present study explores the effects of process parameters, such as welding energy, welding speed, and welding start position, on the post-weld residual stresses. The axial and circumferential residual stress distribution characteristics of the pipe are discussed. The research conclusions reached in this paper are as follows:

(1) The simulation method and experimental method yield similar axial and circumferential residual stress distributions, with an overall average deviation of no more than 30 MPa. The two methods can be used interchangeably to some extent. The greater deviation in the value of the transverse residual stress near the arc-striking and ending points of the pipe circumference is due to the presence of a large heat input near the point. This heat input is incorporated too late to be diffused, thus causing a local stress concentration.
(2) Once the energy output reaches a certain threshold, the surrounding structure undergoes stress homogenization as the heat-affected zone expands. The transverse residual stresses reach extremely high values when the input is between 1007.4 and 859.3 J/mm. The longitudinal residual stresses are less affected by the welding energy, but they exhibit inherent periodic fluctuations. Maximum values are achieved at angles between 0 and 30° and between 180 and 210°, while minimum values are obtained at angles around 90–120° and 270–300°.
(3) The higher the welding speed, the higher the residual stresses on the transverse direction. There is no significant change in the stress gradient when the welding speed reaches 6.6 mm/s. The longitudinal residual stress is less effected, and the overall distribution trend of residual stress is not directly related to the welding speed.
(4) Different interlayer welding start positions can affect the cyclic phase angle of the transverse residual stress distribution in the seam center, but they do not alter its cyclic pattern. This can have an impact on the amplitude and distribution of the longitudinal residual stress along the circumference, while the surface residual stress distribution is homogenized and improved at 120°.

## Figures and Tables

**Table 1:** Chemical composition (wt-%) of base material 316L and welding wire ER316L.

Material	C	Mn	Si	S	P	Cr	Ni	Mo	Fe
Base material	0.025	1.19	0.64	0.011	0.015	17.13	12.57	2.12	Bal.
Welding wire	0.025	1.91	0.42	-	-	19.10	12.58	2.57	Bal.

**Table 2:** Welding process parameters.

Welding Parameter	Root Pass	Filling Passes/Cover Pass
Number of welding layers	1	3
DC/Pulse	DC	AC
Welding current (A)	130	145
Welding voltage (V)	40	40
Welding speed (m/min)	0.06	0.054
Oscillating form	None	Triangular weaving
Oscillation parameters (mm)	None	1.2

**Table 3:** Shape parameter settings for a double ellipsoidal heat source model.

Wall Thickness/*mm*	Bead	$c_{h}/mm$	$c_{p}/mm$	a/mm	b/mm
8	1	2	4	2.5	2
	2–4	2.5	4.5	3	2

**Table 4:** Setting of simulation model parameter comparison.

Comparison Group	Simulated Specimen	Test Program	Welding Layer	Starting Positions of Interlayer Welding (°)	Current	Voltage	Welding Energy (J/mm)	Welding Speed (mm/s)
Group a	168–8 mm	*a*_1_	1	0	130	40	693.3	6
			2–4		170	40	1007.4	5.4
		*a*_2_	1	0	130	40	693.3	6
			2–4		145	40	859.3	5.4
		*a*_3_	1	0	130	40	693.3	6
			2–4		130	40	693.4	5.4
		*a*_4_	1	0	130	40	693.3	6
			2–4		110	40	586.7	5.4
Group b	168–8 mm	*b*_1_	1	0	130	40	693.3	6
			2–4		189	40	773.6	7.8
		*b*_2_	1	0	130	40	693.3	6
			2–4		145	40	773.6	6
		*b*_3_	1	0	130	40	693.3	6
			2–4		131	40	773.6	5.4
		*b*_4_	1	0	130	40	693.3	6
			2–4		102	40	773.6	4.2
Group c	168–5 mm	*c*_1_	1	0	130	40	693.4	6
			2–3		145	40	859.3	5.4
		*c*_2_	1	60	130	40	693.4	6
			2–3		145	40	859.3	5.4
		*c*_3_	1	120	130	40	693.4	6
			2–3		145	40	859.3	5.4

## Data Availability

The data that support the findings of this study are available from the corresponding author upon reasonable request.
